# Rorqual Lunge-Feeding Energetics Near and Away from the Kinematic Threshold of Optimal Efficiency

**DOI:** 10.1093/iob/obab005

**Published:** 2021-03-16

**Authors:** J Potvin, D E Cade, A J Werth, R E Shadwick, J A Goldbogen

**Affiliations:** 1 Department of Physics, Saint Louis University, St. Louis, MO 63103, USA; 2 Institute of Marine Sciences, University of California Santa Cruz, Sant Cruz, CA 95060, USA; 3 Department of Biology, Hampden-Sydney College, Hampden-Sydney, VA 23943, USA; 4 Department of Zoology, University of British Columbia, Vancouver, BC V6T 1Z4, Canada; 5 Hopkins Marine Station, Stanford University, Pacific Grove, CA 93950, USA

## Abstract

Humpback and blue whales are large baleen-bearing cetaceans, which use a unique prey-acquisition strategy—lunge feeding—to engulf entire patches of large plankton or schools of forage fish and the water in which they are embedded. Dynamically, and while foraging on krill, lunge-feeding incurs metabolic expenditures estimated at up to 20.0 MJ. Because of prey abundance and its capture in bulk, lunge feeding is carried out at high acquired-to-expended energy ratios of up to 30 at the largest body sizes (∼27 m). We use bio-logging tag data and the work-energy theorem to show that when krill-feeding at depth while using a wide range of prey approach swimming speeds (2–5 m/s), rorquals generate significant and widely varying metabolic power output during engulfment, typically ranging from 10 to 50 times the basal metabolic rate of land mammals. At equal prey field density, such output variations lower their feeding efficiency two- to three-fold at high foraging speeds, thereby allowing slow and smaller rorquals to feed more efficiently than fast and larger rorquals. The analysis also shows how the slowest speeds of harvest so far measured may be connected to the biomechanics of the buccal cavity and the prey’s ability to collectively avoid engulfment. Such minimal speeds are important as they generate the most efficient lunges.

Sommaire

Les rorquals à bosse et rorquals bleus sont des baleines à fanons qui utilisent une technique d’alimentation unique impliquant une approche avec élan pour engouffrer de larges quantités de plancton et bancs de petits poissons, ainsi que la masse d’eau dans laquelle ces proies sont situés. Du point de vue de la dynamique, et durant l’approche et engouffrement de krill, leurs dépenses énergétiques sont estimées jusqu’à 20.0 MJ. À cause de l’abondance de leurs proies et capture en masse, cette technique d’alimentation est effectuée à des rapports d’efficacité énergétique (acquise -versus- dépensée) estimés aux environs de 30 dans le cas des plus grandes baleines (27 m). Nous utilisons les données recueillies par des capteurs de bio-enregistrement ainsi que le théorème reliant l’énergie à l’effort pour démontrer comment les rorquals s’alimentant sur le krill à grandes profondeurs, et à des vitesses variant entre 2 et 5 m/s, maintiennent des taux de dépenses énergétiques entre 10 et 50 fois le taux métabolique basal des mammifères terrestres. À densités de proies égales, ces variations d’énergie utilisée peuvent réduire le rapport d’efficacité énergétique par des facteurs entre 2x et 3x, donc permettant aux petits et plus lents rorquals de chasser avec une efficacité comparable à celle des rorquals les plus grands et rapides. Notre analyse démontre aussi comment des vitesses d’approche plus lentes peuvent être reliées à la biomécanique de leur poche ventrale extensible, et à l’habilitée des proies à éviter d’être engouffrer. Ces minimums de vitesses sont importants car ils permettent une alimentation plus efficace énergétiquement.

## Introduction

**Fig. 1:**
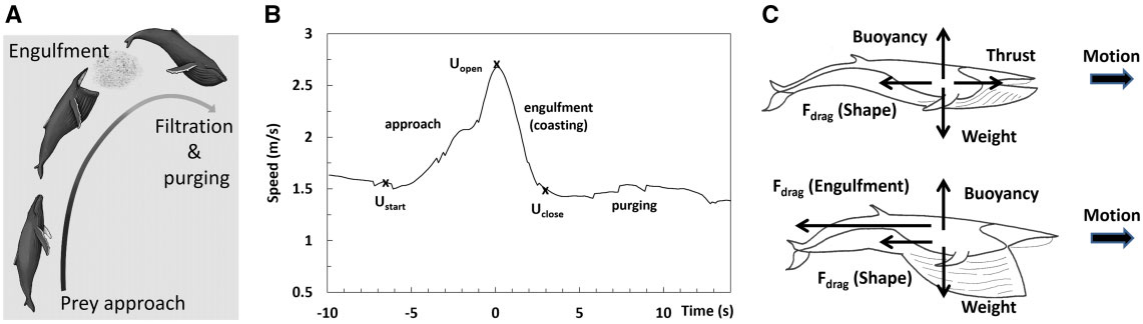
Stages of lunge-feeding near the surface (**A**). (Diagram adapted and used with permission from A. Boersma). Tag-measured speed profile by an 8 m humpback whale (Cade et al 2016), showing the swim speeds at the beginning of prey approach (*U_start_*), the end of approach and onset of mouth opening (*U_open_*), and moment of mouth closure (*U_close_*). The kinematics and the mouth opening and closure occurrences shown in this frame is reflective of the majority of the krill-feeding lunge profiles analyzed in Cade et al 2016 (**B**). Forces on a on a whale during prey approach (top); and when coasting during engulfment (bottom), while using the momentum built-up during approach (Potvin et al. 2020) (C).

At body sizes ranging from 10 m to 30 m, baleen whales such as the humpback (*Megaptera novaeangliae*) and blue whales (*Balaenoptera musculus*) find themselves among the largest vertebrates to inhabit today’s oceans (Marx et al. 2016; Goldbogen and Madsen 2018; Goldbogen et al. 2017, 2019a). As members of the rorqual family (Balaenopteridae), they have evolved morphologies adapted for lunge feeding, a prey-acquisition strategy that enhances energy collection from ephemeral and patchy food resources, while doing so most efficiently at larger body sizes (Goldbogen and Madsen 2018; Goldbogen et al. 2019a; Potvin et al. 2020; Fig. 1). With rorquals foraging in the productive waters of the globe’s temperate zones in spring and summer, high feeding efficiencies are necessary for the accumulation of large fat reserves needed for the fasting that occurs during fall and winter when migrating to, and breeding in, the tropics hundreds (Abrahms et al. 2019) or thousands of miles away (Bailey et al. 2009; Horton et al. 2011; Owen et al. 2017).

Rorquals are edentulous filter-feeders that forage on large aggregations of small prey, typically plankton (krill; 10–40 mm) or schools of forage fish (e.g., anchovies and capelin; 10–20 cm; Doniol-Valcroze et al. 2011; Cade et al. 2016, 2020; Goldbogen et al. 2017; Guilpin et al. 2020). As lunge feeders, they accelerate while approaching the prey, to subsequently engulf it in large numbers along with the water in which it is embedded (Fig. 1; Goldbogen et al. 2017; Potvin et al. 2020). This is followed by prey retention and water expulsion out of their then-inflated buccal cavity via baleen filtration (Fontaine 2007; Goldbogen et al. 2017; Werth et al. 2018; Potvin et al. 2020). Although both fish- and krill-feeding take place at the surface, foraging on krill is also carried out at depths exceeding 100 m where patches of greater density can be found, and in sizes large enough to enable several lunges during a single dive (as many as 5–20 lunges, depending on species; Fig. 1; Goldbogen et al. 2011, 2012; Friedlaender et al. 2019; Guilpin et al. 2019).

One metric of foraging performance is feeding efficiency (*FE*), a ratio of the chemical energy *E_prey_* made available from the ingested prey, to the (metabolic) energy *E_expend_* spent by a whale to capture the prey:

(1) $$FE\equiv \frac{E_{\text{prey}}}{E_{\text{expend}}}$$

Expenditures generally include those incurred during an entire feeding dive, that is, during descent to the patch, execution of multiple lunges, and finally, ascent to and, then, recovery at the surface (Goldbogen et al. 2011). Collecting prey at an efficiency equal to unity implies an energetically neutral harvest. However, as capital breeders (Christiansen et al. 2013) rorquals need significantly greater efficiencies to yield the energetic surpluses that can be stored in the fat reserves used during migration and breeding.

Rorquals achieve extraordinary levels of efficiency, namely up to *FE* = 10–30 (Goldbogen et al. 2011, 2012, 2017, 2019a; Guilpin et al. 2019), which well-exceed those of land carnivores such as lions (*FE* = 3; Williams and Yeates 2004). This performance depends on biological factors—mostly expressed in the numerator of Equation (1)—such as the prey’s spatial availability and energy density, as well as its ability to escape an approaching predator (Goldbogen et al. 2017, 2019a). Another significant biological factor is a whale’s oropharyngeal cavity morphology and size, which determine how much of the prey can be captured in a single gulp (Fig. 1; Goldbogen et al. 2010, 2017, 2019a; Werth and Ito 2017; Cade et al. 2020; Shadwick et al. 2019). In comparison to single-prey item foraging by the toothed whales (dolphins, orcas, sperm whales, etc.), all baleen whales—including balaenid whales—bulk-forage on plankton aggregations and at significantly higher feeding efficiencies, thereby enabling evolution to significantly greater body sizes in extant species (Goldbogen et al. 2019a). Evidently, access to large sources of high energy density prey (albeit patchy), coupled with a capability to capture enormous quantities of it in a single gulp, reduce expenditures as compared with raptorial searching and chasing of individual prey items.

On the other hand, and per the equation’s denominator, the rorquals’ high *FE* also depends on their capacity to carry out a feeding lunge at relatively low costs, that is, as incurred by the musculature of the fluking tail used for propulsion, and by muscle embedded in the ventral skin’s elastin matrix during engulfment (also known as Ventral Groove Blubber—VGB; Orton and Brodie 1987; Fontaine 2007). The latter is necessary to push forward the prey-water mixture to the speed of the whale from a state of rest (Potvin et al 2009, 2020; Goldbogen and Madsen 2018; Goldbogen et al. 2019a).

Muscle use is closely linked to the manner in which engulfment is carried out. In krill-feeding lunges, bio-logging sensor kinematics (Cade et al. 2016) and drone footage (Torres et al. 2020) suggest most large rorquals to engulf the prey and water while decelerating from a state of high velocity to one of low but non-zero velocity (Fig. 1), in a mode denoted here as *coasting engulfment* (Potvin et al. 2020). This contrasts with engulfing while fluking— that is, as in a *powered engulfment* scenario—in which a whale completes at least part of the engulfment cycle while accelerating (Potvin et al. 2012; Simon et al. 2012; Cade et al. 2020). Coasting engulfment is a low energy approach to lunge feeding, in which the accelerative motions are carried out at low drag with the mouth closed while approaching the prey. In contrast, and unless carried out at low speed (Cade et al. 2020), fluking with the mouth open will be energetically costly due to its associated high drag. Herein, the focus shall be on the coasting engulfment of krill rather than fish (powered or coasting), a case further discussed in Cade et al. (2020).

**Fig. 2:**
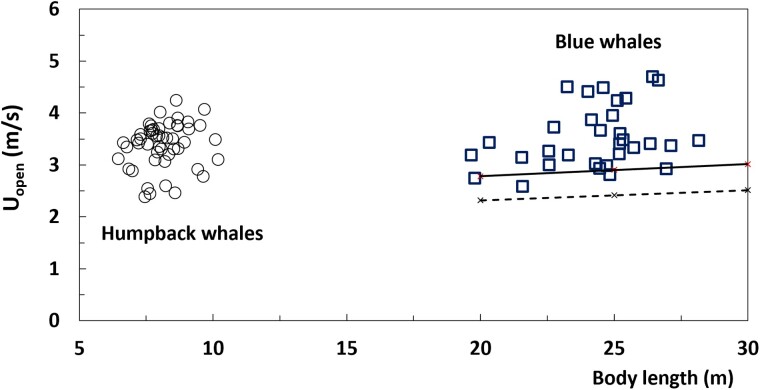
Bio-logging sensor tag-measured swim speed at mouth opening (*U_open_*) versus estimated body lengths, both from Cade et al. (2016). All data for krill–feeding by rorquals at depth. The minimal speeds (*U_min_*) lines are calculated from Equation (12), in the cases of the maximal net approach speeds of 1.0 m/s (dashed lines) and 1.2 m/s (continuous).

Coasting engulfment is essentially an inelastic collision between a whale and its (to be) engulfed mass in which the allometric increases of a whale’s buccal cavity volume leads to a capping of the whale’s initial momentum lost to the water (Potvin et al. 2020). A reduction in the mass-specific muscular force follows from the kinematics of repeated lunges at depth (Fig. 1) in which larger sizes entail longer engulfment durations. Herein, the energetic impact of this lunge-feeding mode is brought to the fore, with the use of the more generally applicable work-energy theorem (an integration of Newton’s second law of motion; Potvin et al. 2010; Goldbogen et al. 2011, 2019a), rather than with time-dependent hydrodynamical modeling (Potvin et al. 2009, 2012; Goldbogen et al. 2011, 2012). Focusing on the cases of humpback and blue whales that use coasting engulfment at depth, and using the kinematics collected during a recent bio-logging tag campaign (Cade et al. 2016), the expenditures are shown to become low enough to yield high efficiency particularly at larger body sizes (Goldbogen et al. 2019a). On the other hand, and with humpback and blue whales of various sizes documented to approach krill over the same range of speeds (2–5 m/s; Fig. 2; Doniol-Valcroze et al. 2011; Goldbogen et al. 2011; Cade et al. 2016; Guilpin et al. 2019; Torres et al. 2020), we also show how high-efficiencies could be drastically reduced at fast foraging speeds regardless of size; and as a corollary, how slow and small rorquals could feed at efficiencies similar to those of the largest rorquals feeding at high speeds. This follows from the significant rises in expenditure originating whenever the swimming speeds of approach and corresponding increases in kinetic energy are higher (proportional to velocity squared); and also, to the greater ventral cavity wall mechanical work needed to push the engulfed prey-water mixture forward and to higher speeds.

This article is organized as follows. The next section reviews the use of the work-energy theorem for calculation of lunge energetics during coasting engulfment, along with its conversion into metabolic expenditures. A novel approach to the computation of the drag work associated with the flows moving past the body during engulfment is discussed as well. The calculated expenditures and efficiencies incurred by tagged blue (22 m, 27 m) and humpback (8 m) whales (Cade et al 2016) are presented in *Results*, along with sensitivity calculations connected to the uncertainties in body mass, frictional drag, and body expenditures incurred by energy-producing sites external to the musculature of the tail and VGB. Other aspects necessary for energy scaling analysis are analyzed as well, particularly with regards to speed-scaling of lunge feeding durations, and to the prediction of the speeds incurring the smallest expenditures and the highest efficiencies (at fixed prey density). Following the results, the *Discussion* shows how expenditure and efficiency speed-scaling arise and imply partial cancelation of the benefits of large body size at high foraging speeds. This article ends with *Concluding Remarks*, followed by an Appendix of mathematical derivations.

## Materials and methods

### The forces at play

The work performed by the musculature of a whale’s tail or buccal cavity is a reflection of the external forces applied to the body (Fig. 1; Potvin et al. 2009, 2012, 2020). Body weight (W) and buoyancy (B) turn out to be unimportant here as they nearly cancel each other out near the surface, where buoyancy is effected by body density and lung volume expansion (Miller et al. 2016); or compensated at depth (beyond 60–100 m) by the lift generated by the tilting of the foil-shaped flippers (Cooper et al. 2008). More important is the fluking thrust and drag generated in both mouth-open and -closed configurations. During engulfment, the so-called *engulfment drag* is generated in reaction to the forward push of the engulfed mass by musculature embedded in the VGB (per Newton’s third law; Potvin et al. 2009). For large rorquals, this force is more likely to be muscle-based rather than elastic, given the low levels of elastin matrix stretching observed during feeding (Shadwick et al 2013). Finally, there is the other drag component, namely, *shape drag*, generated by flows moving externally to the body during both approach to the prey and its engulfment (Potvin et al. 2009, 2020). (Note that a potential coupling between shape and engulfment drag, likely mediated by the pressure differences between the inner and outer buccal cavity wall, has been ignored herein).

### Mechanical expenditures of a lunge

Expenditure calculation is a two-step process in which the mechanical work (*W*) performed by relevant muscle groups is first estimated from the work-energy theorem (Potvin et al. 2010; Goldbogen et al. 2011). This is followed by a calculation of the corresponding metabolic energy spent by those muscle structures and elsewhere in the body. In previous studies, the energetics was obtained from simulations of time-dependent engulfment forces expressed in parametric form and based on assumed rates of the mouth opening (Potvin et al. 2009, 2012; Goldbogen et al. 2011, 2012). A simpler, yet more general alternative is used here, made possible by lunge-feeding being a sequence of highly accelerated motions (Fig. 1B) for which the energetics becomes dominated by attendant changes in kinetic energy rather than the frictional drag losses that usually dominate non-feeding transport.

The mechanical expenditures of prey-approach and coasting engulfment are evaluated separately. The corresponding equation of motion for the former is *M_body_ a = Thrust—$F_{D}^{\text{shape}}$* (Fig. 1) which, after integration over traveled distance, yields the following energy budget (Goldbogen et al. 2019a):

(2) $$\begin{array}{c} \frac{1}{2}M_{\mathrm{body}}\left(U_{\mathrm{open}}{}^{2}-U_{\mathrm{start}}{}^{2}\right)=W_{\mathrm{flukes}}-W_{\mathrm{drag}}^{\mathrm{shape}}\\ =W_{\mathrm{flukes}}-W_{\mathrm{drag}}^{\mathrm{parasite}}-\frac{1}{2}kM_{\mathrm{body}}\left(U_{\mathrm{open}}{}^{2}-U_{\mathrm{start}}{}^{2}\right) \end{array}$$

The speeds *U_start_* and *U_open_* are a whale’s forward velocity at the beginning and end of the acceleration respectively, and available from bio-logging tag data (Goldbogen et al. 2011; Cade et al 2016; Fig. 1). Parameter *M_body_* is the mass of the body, available from morphological data obtained from strandings and industrial whaling (Kahane-Rapport and Goldbogen 2018). In krill-feeding lunges, tag data suggest *U_start_* as being close to the whale’s speed at mouth closure (*U_start_* = *U_close_*; Doniol-Valcroze et al. 2011; Cade et al. 2016). As explained in the Appendix, the last two terms in the second line in Equation (2) express shape drag as the sum of a “parasite” component corresponding to the viscous friction and pressure drag acting near the body surface and its boundary layer (Goldbogen et al. 2015; Potvin et al. 2020); and of an “acceleration reaction” force accelerating the fluid above that layer and explicitly over time (resulting in coefficient *k*; Lamb 1932; Pope 1951; Newman 1977; Denny 1993; Potvin et al. 2020). Both drag sources are calculated from Equation (A6), which is a very approximate scheme, but of secondary importance in comparison to the kinetic energy variations incurred (left-hand-side [LHS] of Equation 2). Omitting such drag sources generally underestimates the locomotor expenditures, with small errors in the case of the larger rorquals (*L_body_ >*12 m), but more significant errors at smaller sizes, as further discussed below.

A similar treatment is applied to the engulfment stage, where *M_body_* a = Thrust—*F_D_^shape-^F_D_^engulf^*. Integration over travel distance leads to the work carried out by VGB musculature, -Δ${\text{W}}_{\text{VGB}}^{\text{engulf}}$, estimated as follows (with Δ${\text{W}}_{\text{VGB}}^{\text{engulf}}$ denoting the absolute value of the work; Potvin et al. 2010, 2020):

(3) $$\begin{array}{c} \frac{1}{2}M_{\mathrm{body}}\left(U_{\mathrm{close}}{}^{2}-U_{\mathrm{open}}{}^{2}\right)\approx -W_{\mathrm{VGB}}^{\text{engulf}}+\\ (-1)\left\{\left[M_{\mathrm{body}}\left(U_{\mathrm{open}}-U_{\mathrm{close}}\right)-M_{\mathrm{water}}U_{\mathrm{close}}\right]\cdot \frac{1}{2}\left(U_{\mathrm{open}}+U_{\mathrm{close}}\right)\right\} \end{array}$$

The terms in the curly brackets are new and correspond to an approximation of the shape drag work done by the frictional, near wake and acceleration reaction (Appendix). Those are to again become more significant only at smaller body sizes, as shown in *Results*. Neglecting such terms may overestimate or underestimate the VGB expenditures depending on the value of *U_close_*. As with prey approach, and mostly as a body surface effect, shape drag work remains significantly smaller than the body volume-dependent kinetic energy variations (LHS Equation 3), particularly at large body size.

Parameter *M_water_* is the mass of the engulfed water-prey mixture, calculated by approximating the filled buccal cavity as two juxtaposed quarter-ellipsoids spanning the skull’s width (*w_skull_*), mandible length (*L_mandible_*) and VGB length (*L_VGB_*); and from adjustment factor Ψ (∼1.03–1.14) and seawater mass density *ρ_water_* (Goldbogen et al. 2007, 2010; Potvin et al. 2012, 2020):

(4) $$M_{\text{water}}=\rho_{\mathrm{water}}\cdot \Psi \left[\frac{\pi }{3}L_{\text{VGB}}L_{\text{mandible}}\frac{1}{2}w_{\text{skull}}\right].$$

Although Equation (3) applies to balaenopterids of all sizes, special attention is devoted here to the larger, engulfment drag-dominated blue whales (Goldbogen et al. 2019a; Potvin et al. 2020). This allows treating coasting engulfment as a perfectly inelastic collision between a whale and to-be engulfed mass, a process for which the final and initial speeds are related as (Potvin et al. 2020):

(5) $$\frac{U_{\text{close}}^{}}{U_{\text{open}}^{}}=\left(\frac{M_{\mathrm{body}}}{M_{\mathrm{body}}+M_{\text{water}}}\right).$$

Using Equation (5) in (3) results in a simpler expression for the work done by VGB musculature, namely:

(6) $$\Delta W_{\mathrm{VGB}}^{\text{engulf}}\approx \frac{1}{2}M_{\mathrm{body}}U_{\mathrm{open}}{}^{2}\left[1-{\left(\frac{M_{\text{body}}}{M_{\text{body}}+M_{\mathrm{water}}}\right)}^{2}\right]$$

Note that Equation (6) also follows after using Equation (5) and neglecting shape drag altogether (Goldbogen et al. 2019a). Inserting Equation (5) in (2) (with *U_start_ = U_close_*) yields a similarly simple result, meaning that the work done during both prey approach and engulfment is proportional to parameter ${\text{U}}_{\text{open}}^{2}$.

It should be noted that in most of the krill-feeding lunges analyzed in Cade et al. (2016), the mouth opens near the maximal speeds of prey approach (*U_max_=U_open_*), that is, before starting its no-fluking deceleration. This is the scenario discussed here. On the other hand, they have also documented several instances of lunges occurring with the mouth opening only halfway through the decelerative stage following the acceleration. In other words, these are lunges in which prey approach incorporates both accelerative and decelerative kinematics, but with engulfment remaining as purely decelerative. Equations (2) and (3) are still valid here, that is, with Equation (2) describing the energetics of the accelerative portion of prey approach with speeds *U_start_* and *U_max_ > U_open_*; and Equation (3), that of the mouth-opening and -closing portions of the decelerative stage characterized (again) by speeds *U_open_* and *U_close_*, that is, as long as fluking is not occurring during any portion of this stage (Where fluking occurs, extra terms must be added in Equation (3), as was done in the analysis of fish-feeding lunges by Cade et al. (2020)).

### From the mechanical to the metabolic

The relationship between the expended mechanical and metabolic energies is approximated by dividing the work by a metabolic efficiency constant (*η_metab_*), that is, in cases where the mechanical work is important to the motion changes at hand (Blake 1983; Pennycuick, 1992):

(7) $$E_{\text{metab}}|{}_{\mathrm{VGB}}=\frac{W_{\mathrm{drag}}^{\mathrm{engulf}}}{\eta_{\text{metab}}}$$

(8) $$E_{\text{metab}}|{}_{\text{fluking}\;\text{tail}}=\frac{W_{\mathrm{flukes}}}{\eta_{\text{prop}}\eta_{\text{metab}}}$$

The metabolic efficiency constant *η_metab_* is set at 0.25 to reflect the 4 Joules of muscle chemical energy spent to generate 1 Joule of mechanical energy while losing 3 Joules in heat. Using efficiency constants is an approximation and one that has never been validated in large animals. Note also that Equation (8) includes the additional “propeller” efficiency constant (*η_prop_*) to account for the energy used by the flukes to move fluids in directions orthogonal to that of propulsion (Webb 1971; Fish 1993, 1998). With cetaceans, η_prop_ = 0.7 to 0.8, depending on fluke design (Fish and Rohr 1999).

Finally, body expenditure calculation necessitates the addition of the metabolic energy generated by all chemical energy-consuming sites used during a lunge by organs and tissue external to the fluking tail and VGB musculature. This term is estimated from a so-called *ceteral* expenditure rate (“*cetER”*; from the Latin *cetera*, “rest of”), multiplied by a lunge’s duration (*T_lunge_*) incorporating the prey-approach (*T_appr_*), engulfment (*T_engulf_*), and water expulsion/filtration (*T_filter_*; all available from bio-logging tag data):

(9) $$E_{1-\text{lunge}}=E_{\text{metab}}|{}_{\mathrm{VGB}}+E_{\text{metab}}|{}_{\text{fluking}\;\text{tail}}+\mathrm{cetER}\cdot \left(T_{\mathrm{appr}}+T_{\mathrm{engulf}}+T_{\mathrm{filter}}\right)$$

By definition, ceteral expenditures are likely to remain out of the realm of direct measurement, with their value intimately connected to the amounts and rates of tail/VGB use in real time. Moreover, these should not be confused with metabolic rates of the entire body (basal, resting or active) which here are estimated from the ratio *E_1-lunge_/T_lunge_*.

Interestingly, ceteral expenditures can be bounded from above by assuming the combined tail and VGB expenditures as greater, that is, $\mathrm{cetER}\cdot T_{\mathrm{lunge}}<E_{\text{metab}}|{}_{\mathrm{VGB}}+E_{\text{metab}}|{}_{\text{fluking}\;\text{tail}}$. This hypothesis is motivated by blue whale bradycardia, a physiological response presumably fueled by local muscle oxygen store depletion rather than by blood oxygen transport and depletion (Goldbogen et al. 2019b). Furthermore, the bound can be expressed in terms of a well-known basal metabolic expenditure formula (Hemmingsen 1960; Kleiber 1975), that is, with $\mathrm{cetER}\cdot T_{\mathrm{lunge}}\equiv f\cdot 4.1\cdot M_{\mathrm{body}}{}^{0.75}$ and extra proportionality factor *f*. From the above inequality one arrives at

(10) $$\frac{E_{\text{metab}}|{}_{\mathrm{VGB}}+E_{\text{metab}}|{}_{\text{fluking}\;\text{tail}}}{4.1M_{\text{body}}{}^{{}^{0.75}}T_{\text{lunge}}}\equiv f^{\mathrm{upper}}\geq f,$$

an expression readily estimated from tag data and Equations (2) to (8). Interestingly, this upper bound scales with ${\text{U}}_{\text{open}}^{2}$ per Equations (2) to (6), a velocity-sensitive trend expected with increasing muscular effort. From the inputs discussed in the next section, one arrives at ceteral rates bounded at about *f^upper^* = 1.4–2.7 depending on lunge duration, swim speed and body size.

Note that Equation (9) also neglects the calculation of the energy spent by the VGB muscle to contract the ventral skin and blubber during the water expulsion/filtration stage, as currently out of reach of calculation. Such contraction has significantly longer duration as compared to engulfment (10–20 times longer; Goldbogen et al. 2011, 2012; Potvin et al. 2020) and is likely a low-power process by VGB musculature.

### Captured prey energy

With the energetic expenditure known, the *FE* ratio (Equation 1) is calculated after estimating the prey energy acquired (per lunge) via (Goldbogen et al. 2011):

(11) $$E_{\text{prey}}{}^{1-\text{gulp}}=0.84\varepsilon_{\mathrm{krill}}\rho_{\mathrm{prey}}\left(\frac{M_{\mathrm{water}}}{\rho_{\mathrm{water}}}\right)$$

Parameter *ε_krill_* is the energy density of a kilogram of krill (∼4600 kJ/kg; Goldbogen et al. 2011, 2012), corrected by the factor 0.84 to account for the energy lost to digestion & excretion (Goldbogen et al. 2011). *ρ_prey_* is the patch’s prey mass density expressed in krill mass per unit volume of ocean, that is, pre-harvest and assuming near-100% catch levels (as suggested by drone video; see for example Torres et al. 2020). The ratio in parenthesis is the volume of the whale’s inflated buccal cavity, calculated from the quotient of the engulfed mass over seawater density (Equation 4 and Table 1).

**Table 1:** Morphology and kinematics

	Humpback whale	Blue whale	Blue whale	Remarks
*M_body_* (kg)	8000 (8000)	67,273 (23,991)	129,005 (46,081)	Kahane-Rapport and Goldbogen (2018; blue whale); and Potvin et al. (2012; humpback whale)
L (m) Bio-logging Tag Number	8.00 mn160727-11	22.72 bw160224-8	27.40 bw160727-10	Cade et al. (2016)
*L_VGB_* (m)	4.31 (0.12)	12.99 (0.26)	16.36 (0.32)	Kahane-Rapport and Goldbogen (2018)
*L_jaw_* (m)	1.62 (0.13)	4.34 (0.31)	5.65 (0.41)	Kahane-Rapport and Goldbogen (2018)
*W_head_* (m)	1.32 (0.16)	2.61 (0.15)	3.27 (0.19)	Kahane-Rapport and Goldbogen (2018)
*S_wet_* (m^2^)	27.5 (N/A)	109.9 (N/A)	167.9 (N/A)	Fish (1993, 1998)
*U_open_* (m/s)	3.56 (0.26)	2.78 (0.13)	3.25 (0.33)	Cade et al. (2016) and Fig. 3
*M_water_* (kg)	4982 (290)	90,350 (1648)	185,595 (3446)	Equation (4)
*U_close_* (m/s)	2.19 (0.55)*	1.19 (0.15)*	1.33 (0.36)*	*Equation (5)
	0.70**	0.70**	0.70**	**Variations suggested by recent tag data (W. Gough, pers. comm.)
	1.50**	1.50**	1.50**	
*U_min_* (m/s)	1.95 (NA)	2.81 (NA)	2.93 (NA)	Equation (12)
Engulfment time (s)	1.18 (0.16)	5.65 (0.45)	6.58 (0.76)	Cade et al. (2016)
Prey-approach time (s)	16.5 (8.9)	13.5 (3.9)	19.5 (10.0)	Cade et al. (2016)
Purging time (s)	27.5 (3.7)	61.9 (12.2)	48.8 (9.6)	Cade et al. (2016)
Lunge duration (s)	45.1 (9.6)	81.0 (12.8)	74.9 (13.9)	Summation of the above three durations

Body length, swim speed at mouth opening and lunge stage durations were measured and averaged over lunges (Cade et al. 2016). All other parameters estimated from the indicated references. SDs are shown in parentheses and were obtained from analysis of the tag data (Cade et al. 2016) and a morphology database (Kahane-Rapport and Goldbogen 2018); and “N/A” when unavailable.

## Results

### Three case studies of lunge-feeding energetics

Tables 2 and 3 show the expenditures calculated from the morphology and kinematics listed in Table 1 in the cases of humpback (8 m) and blue whales (22 m and 27 m) tagged by Cade et al. (2016). Here, body lengths were inferred from the width of the VGB furrow stretching gaps observed in tag-borne video of the buccal cavity rather than from direct drone-based photogrammetry.

**Table 2:** Metabolic energy expenditures: fluking vs. engulfment stages; vs. shape drag

Specimen/input and output data	Humpback whale	Blue whale	Blue whale	Source
L (m)	8	22.73	27.40	Cade et al. (2016)
Bio-logging tag number	mn160727-11	bw160224-8	bw160727-10	Cade et al. (2016)
*U_open_* (m/s)	3.56 (0.26)	2.78 (0.13)	3.25 (0.33)	Cade et al. (2016) and Fig. 3; SD from tag data.
*U_close_* (m/s)	2.19 (0.55)	1.19 (0.15)	1.33 (0.36)	Equation (5); SD from mass data
	126	875	2267	Equations (6) and (7); *U_close_* from Equation (5)
*E_metab_ǀ_VGB_* (kJ)	76	1171	2644	Equations (3) and (7); *U_close_* = 1.5 m/s
	30	445	1026	Equations (3) and (7); U_close_ = 0.7 m/s
*E_metab_ǀ_fluking_* (kJ)	157	1094	2834	Equations (2), (A6), and (8); *U_start_* = *U_close_* (Equation (5); $\tilde{F}$= 0 (no shape drag)
				*U_start_* = *U_close_* = 1.5 m/s; $\tilde{F}$= 2
	348	1183	3324	*U_start_* = *U_close_* = 0.7 m/s; $\tilde{F}$= 2
	352	1426	3752	
*E_metab_ǀ_fluking_* + *E_metab_ǀ_VGB_* (kJ)	283	1969	5101	Sum of the previous two rows. Equals *E_1-lunge_* in Equation (9) when setting *cetER* = 0.
	424	2354	5968	
	382	1871	4778	
				Values above, divided by engulfment duration (rounded).
				*U_start_* = *U_close_* (Equation 5)
*P_metab_ǀ_VGB_* (kW)	112	157	324	*U_start_* = *U_close_* = 1.5 m/s
	68	210	378	*U_start_* = *U_close_* = 0.7 m/s
	27	80	147	
				Values above, divided by prey approach duration (rounded).
*P_metab_ǀ_fluking_* (kW)	9	79	142	*U_start_* = *U_close_* (Equation 5)
	20	86	166	*U_start_* = *U_close_* = 1.5 m/s
	21	103	188	*U_start_* = *U_close_* = 0.7 m/s

The morphology and kinematics input are listed in Table 1. Other inputs are *ε_prey_* = 4600 kJ and *ρ_prey_* = 0.18 kg/m^3^ (Equation 11) (Goldbogen et al. 2011); $\tilde{F}$= 0 or 2 (i.e., without or with shape drag, respectively), *ρ* = 1025 kg/m^3^ and *ν*= 1.19 × 10^−6^ m^2^/s, *k_added_* = 0.03 (blue whale) and 0.05 (humpback; Equation A3); *η_metab_* = 0.25 and *η_prop_* = 0.80 (Equation A2); and *f *=* *1.45 (Equation 10). Calculations omitting shape drag are shown in the first row of cells containing triple entries. SDs are shown in parentheses.

**Table 3:** Metabolic energy expenditures for the prey approach, engulfment and purging stages combined

Specimen/input and output data	Humpback whale	Blue whale	Blue whale	Source
L (m)	8	22.73	27.40	Cade et al. (2016)
Bio-logging tag number^10^	mn160727-11	bw160224-8	bw160727-10	Cade et al. (2016)
*U_open_* (m/s)	3.56 (0.26)	2.78 (0.13)	3.25 (0.33)	Cade et al. (2016) and Fig. 3; SD from tag data.
*U_close_* (m/s)	2.19 (0.55)	1.19 (0.15)	1.33 (0.36)	Equation (5); SD from mass data
*cetER* (kW)	5	25	40	*cetER* = *f 4.1 M^0.75^*
*cetER* energy (kJ)	232	2111	3116	Equation (9) previous row times lunge duration
				Equations (9) with previous row with *f *=* *1.45;
Single lunge expenditure *E_1lunge_* (kJ)	515	4080	8217	*U_start_* = *U_close_* (Equation (5)
	656	4465	9085	*U_start_* = *U_close_* = 1.5 m/s
	614	3982	7894	*U_start_* = *U_close_* = 0.7 m/s
				*E_total_*/*T_lunge_*; via Equation (9).
Power (kW) expended during a single lunge	11	48	107	*U_start_* = *U_close_* (Equation (5)
	14	53	118	*U_start_* = *U_close_* = 1.5 m/s
	13	47	103	*U_start_* = *U_close_* = 0.7 m/s
Prey energy (kJ)	3380	61,307	125,937	In a single “gulp” Equation (11)
				Equation (1);
FE (single lunge)	7	14	14	*U_start_* = *U_close_* (Equation (5)
	5	15	15	*U_start_* = *U_close_* = 1.5 m/s
	6	15	16	*U_start_* = *U_close_* = 0.7 m/s

The input values are the same as in Table 2. Calculations omitting shape drag are shown in the first row of cells containing triple entries. SDs are shown in parentheses.

The ceteral term (*cetER T_lunge_ = f 4.1 $M_{\text{body}}^{0.75}$*) calculated in Table 2 was evaluated using *f = 1.45*, as suggested by the lowest value of the upper bounds estimated via Equation (10). Using the fluking tail and VGB expenditures $E_{\text{metab}}|{}_{\mathrm{VGB}}\;\text{and}\;E_{\text{metab}}|{}_{\text{fluking}\;\text{tail}}$ listed in Table 2, along with the lunge durations, mouth open speeds and body mass shown in Table 1, suggest *f^upper^ =* 2.39, 1.45 and 2.72 for the 27 m and 22 m blue and 8 m humpback whales respectively. With *f^upper^* being proportional to ${\text{U}}_{\text{open}}^{2}$, the value *f = 1.45* ensures a calculation that remains consistent with the assumed inequality $E_{\text{metab}}|{}_{\mathrm{VGB}}+E_{\text{metab}}|{}_{\text{fluking}\;\text{tail}}<{\text{cetER}\;T}_{\text{lunge}}$at all speeds, and most importantly, at the lowest of speeds.

Although reproducing expenditures obtained elsewhere (Goldbogen et al. 2011, 2012), Tables 2 and 3 highlight aspects never discussed before, particularly with regard to the relative contributions the prey approach versus engulfment stages, ceteral expenditures, shape drag contributions, etc. Estimated efficiencies are shown as well, but only calculated via Equations (9) and (10) for the execution of a single lunge, that is, minus the descent and ascent stages, and evaluated at a prey density *ρ_prey_* = 0.18 kg/m^3^—a value well-within the range encountered in Monterey Bay, CA (0.63 kg/m^3^ in the mean and 2.5 kg/m^3^ Standard Deviation; Goldbogen et al. 2019a).

Given the large uncertainties associated with *M_body_* (35–100%; Table 1; Kahane-Rapport and Goldbogen 2018; Potvin et al. 2020), the tabulated results are only estimates where mass is setting the overall scale in gained/lost kinetic energy (Equations 2 and 3). On the other hand, the three cases explored here are of sufficiently different size and mass, and the other input known to better accuracies (Table 1), to yield trends beyond errors. As applied to blue whales, the results turn out consistent with previous estimates (Goldbogen et al. 2011; Potvin et al. 2012) after accounting for the differing initial speeds used therein (e.g., *U_open_* = 4 m/s at 27 m body length). Tables 2 and 3 also display three series of calculations illustrating the contributions of shape drag, a previously unaccounted source of systematic error. Generally the differences, that is, ∼10–20% in blue whales and >50% in small humpback whales, are more conspicuous in the expenditure estimates of the locomotor (*E_metab_/_fluking_*) plus VGB (*E_metab_/_VGB_*) musculature (Table 2; *U_close_* > 1 m/s), than in the metabolic total after addition of the commensurate ceteral expenditures (Equation 9). On the other hand, further assessing the effects of the latter can be done by comparing the sum *E_metab_ǀ_fluking_ + E_metab_ǀ_VGB_* in Table 2 (see Equation 9 with *f *=* *0) versus *E_1lunge_* with *f = 1.45* (Table 3), showing a commensurate contribution in the range of 40–50% of the total.

Listing from largest-to-shortest body sizes, that is, 27 m (blue), 22 m (blue), and 8 m (humpback), the total expenditures for a single lunge stand at about *E_total_* = 8, 4 and 0.5 MJ (without shape drag; Table 3). Given the similar *U_open_*-values used in the three cases (≈3 m/s; Table 1), the difference ends up as a clear body-mass effect, seen more clearly when evaluated per kilograms of body mass (“mass-specific expenditures”): namely, 64, 61, and 64 J/kg. Given the durations recorded by the bio-logging tags (Table 1), such energy outputs translate into power levels of 107, 48, and 11 kW (or 0.8, 0.7, and 1.4 W/kg). In comparison with the basal metabolic rates (BMR) measured on land mammals (4.1 ${\text{M}}_{\text{body}}^{0.75}$; Hemmingsen 1960), such outputs come out at 3.8, 2.8, and 3.2-times greater. Feeding efficiencies (single lunge) turn out quite differently with size, however, with *FE* = 14 (both blue whales) versus = 7 (humpback), an effect due to the (allometric) scaling of the engulfed volume (*M_water_/ρ_water_*; Equation 5) increasing faster than *M_body_* at large *L_body_* (Goldbogen et al. 2012, 2019a; Potvin et al. 2020).

Smaller amounts of musculature energy appear expended during the engulfment stage than during prey approach, and again for the 27 m, 22 m, and 8 m cases, respectively: 2.2 MJ (VGB) versus 2.8 MJ (fluking), 0.87 MJ versus 1.1 MJ, and 0.06 MJ versus 0.35 MJ (Table 2; and in the 8 m case, averaging the “with shape drag” values). On the other hand, and because of its shorter duration, the metabolic power required during engulfment follows the opposite trend, namely: 324 kW (VGB) versus 142 kW (fluking), 157 kW versus 79 kW, and 48 kW versus 20 kW; or in terms of land mammals BMR, 12 (VGB) versus 5 (fluking), 9 versus 5 and 14 versus 6. Finally, and compared to surface breaching by humpback whales (Segre et al. 2020), the prey-approach expenditures by our 8 m humpback whale turn out three-times smaller given the lower maximal prey approach speeds reached, that is, as compared with the 6–7 m/s attained just prior to surface breaking.

### Durations of lunge feeding at depth

The bio-energetic modeling requires using several kinematic parameters accessible from tag data, which in turns permits the derivation of the scaling laws necessary for interpolating the model over foraging speed values not sampled by the tags (next section).

**Fig. 3:**
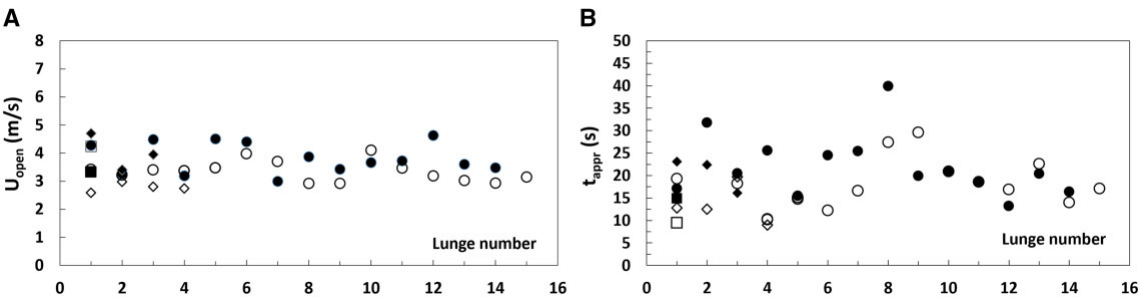
Speed at the end of prey approach (coinciding with *U_open_*) (**A**), and corresponding approach duration (*t_appr_*) (**B**). From Cade et al. (2016). In both frames the “*lunge number*” labels the lunges performed over several successive dives by a given animal: Counting about four lunges per dive in blue whales (Goldbogen et al. 2011), the data for the 27 m individual shown here would characterize 16 lunges carried out over four consecutive dives (see also Potvin et al. 2020). The symbols displayed in both frames correspond to different tag deployments and animals, namely: *bw140820-3b (23.6 m;* filled circles); *bw140806-2 (25.9 m;* filled diamond); *bw140224-8 (22.7 m;* open diamonds); *bw140722-2e (25.7 m;* filled squares); *bw140819-3b (25.1 m;* open squares); *bw160727-10 (27.4 m;* open circles).

**Fig. 4:**
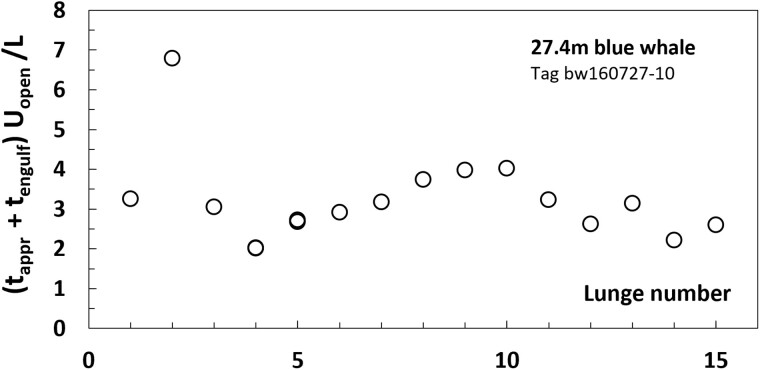
Non-dimensional lunge duration by a given whale and over several dives. Data from Cade et al. (2016). The “lunge number” label is similar to that of Fig. 3.

**Fig. 5:**
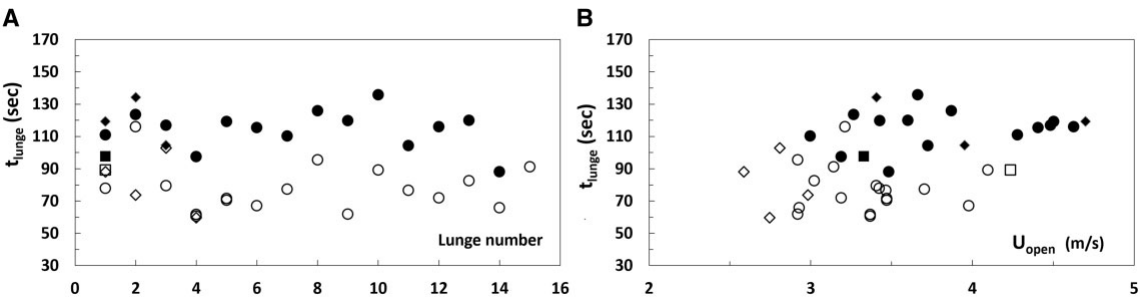
Lunge duration (≡*T_appr_+T_engulf_+T_purge_*) versus lunge number (**A**) and speed (**B**), measured on six tagged blue whales. Data from Cade et al. (2016). In frame (**A**), the “*lunge number*” label is defined in similarity to Fig. 5. In both frames different symbols correspond to different tag deployments and animals, namely: *bw140820-3b (23.6 m;* filled circles); *bw140806-2 (25.9 m;* filled diamond); *bw140224-8 (22.7 m;* open diamonds); *bw140722-2e (25.7 m;* filled squares); *bw140819-3b (25.1 m;* open squares); *bw160727-10 (27.4 m;* open circles).

When plotted versus lunge event (Fig. 3) rather than over body size (Fig. 2), the mouth-open speeds show a surprising degree of regularity over several feeding dives performed by the same individual. Similar trends are seen with the approach and engulfment durations (Fig. 3; Potvin et al. 2020). Combining both with the corresponding *U_open_* into non-dimensional ratios *K* (*≡ time* × *speed/length*) leads to a meaningful averaged value of this parameter (*K = *3 ± 1; Fig. 4). On the other hand, no such scaling law is empirically achievable for lunge duration (*T_lunge_*; Fig. 5), which turns out dominated by the duration of the water expulsion and prey retention stage (*T_purge_*; Goldbogen et al. 2011; Guilpin et al. 2019, 2020; Kahane-Rapport et al. 2020). Here, and intra-specifically (Kahane-Rapport et al. 2020), body size and swim speed appear as secondary factors in determining *T_lunge_*, as compared to other, environmentally and behaviorally driven factors (Goldbogen et al. 2013; Hazen et al 2015; Lesage et al. 2017; Guilpin et al. 2020). Thus, two tag-informed bounding values of the lunge duration are used instead in the upcoming expenditure scaling analysis.

### Minimum speeds for the most efficient harvests

Equations (1)–(3) point to the smallest values in *U_open_* as corresponding to the highest efficiencies (at fixed prey density). Data from bio-logging tags show rorquals of all sizes consistently beginning to engulf krill aggregations at speeds ranging from *U_open_* = 2.5 m/s to about 5 m/s (Fig. 2; Doniol-Valcroze et al. 2011; Goldbogen et al 2011; Cade et al. 2016; Torres et al. 2020). There might be several reasons behind such dispersion, including duration of the water expulsion/filtration stage, patch size in relation to a whale’s size, oxygen management during a dive (Hazen et al. 2015), or limited surface (Lesage et al. 2017; Guilpin et al 2020) or bottom time (Goldbogen et al. 2013). But, other factors may be at play, particularly with regard to the *minimal U_open_*.

Video by drones flying over lunge-feeding at the surface (Torres et al. 2020), along with hundreds of observations from animal-borne cameras (Cade et al. 2016, 2020), show minimal scatter among the krill while attempting to get out of the predator’s way. Why rorquals need to swim so quickly (>2 m/s) to harvest seemingly immobile prey, rather than collecting it at slower speeds, is not well understood. A first explanation may reside with the demonstrated ability by Antarctic krill aggregations (*Euphausia superba*) to detect and cohesively split to avoid nets and submersibles approaching at speeds of up to about 1.0 m/s, but doing so poorly beyond 1.2 m/s (Hamner and Hamner 2000). Rapidly approaching an aggregation may thus arise from the necessity to overwhelm the predator-detection performance of the krill (via visual or pressure wave sensing) and its own limited swimming performance (0.05–0.2 m/s in pleopod or tail-flipping modes; Kils and Marschall 1995; Hamner and Hamner 2000; Murphy et al. 2011), in similarity to lunge feeding on fish (Cade et al. 2020).

Rorqual morphology and tissue physics may also require a minimal speed. Orton and Brodie (1987) estimated one at about *U_open_* = 3 m/s to provide enough internal pressure to unfold and expand the VGB against the elastic stresses characteristic of the stretched VGB found on the bloated bodies of decomposing whales (Shadwick et al. 2013). Such estimated minimal swim speed is unlikely, however, given the significantly lower amounts of VGB stretching (“strains”) observed in actual lunges (Cade et al. 2016; Torres et al. 2020). Additionally, “bloated state” strains are likely to exceed the physiologically sustainable length of the muscle fibers embedded in the VGB (Shadwick et al. 2013). Interestingly, a similar calculation by Orton and Brodie (summarized in their Tables 1 and 2) lowers the minimal “inflation” speed to about 1 m/s when using strain values now inferred from tag-borne video.

Constraining the decelerative motion of coasting engulfment by the large rorquals (Fig. 1) to speeds *(U(t))* above ∼1.2 m/s would limit the speed at mouth closure to *U_close_* ≥ 1.2 m/s as well. Thus, and per Equation (5), a lower bound emerges as

(12) $$U_{\mathrm{open}}\geq U_{\min}=\left(1.2\;m/s\right)\cdot \left(1+\frac{M_{\mathrm{water}}}{M_{\mathrm{body}}}\right).$$

Although the factor 1.2 m/s shown here corresponds to the “maximal net approach speed” mentioned by Hamner and Hamner (2000), higher or lower values can be substituted to reflect the collective escape performance of other plankton of interest after assessment by tow net experiments (Kils and Marschall 1995; Hamner and Hamner 2000). Figure 2 compares Equation (12) for two likely values of this net approach parameter (1.0 m/s and 1.2 m/s), to broadly match the lowest whale speeds (*U_open_* = 3 m/s) detected by tags deployed on blue whales foraging off the coasts of Chile, South Africa, and California (USA; Goldbogen et al. 2011; Cade et al 2016). Moreover, Equation (12) matches the observed weak dependence over body length (Fig. 2), namely, by changing only slightly from *L_body_ =* 20 m–30 m per current morphology scaling (Kahane-Rapport and Goldbogen 2018). This is an interesting trend which should be verified in future tag-based studies.

### Energy scaling with respect to speed

**Fig. 6:**
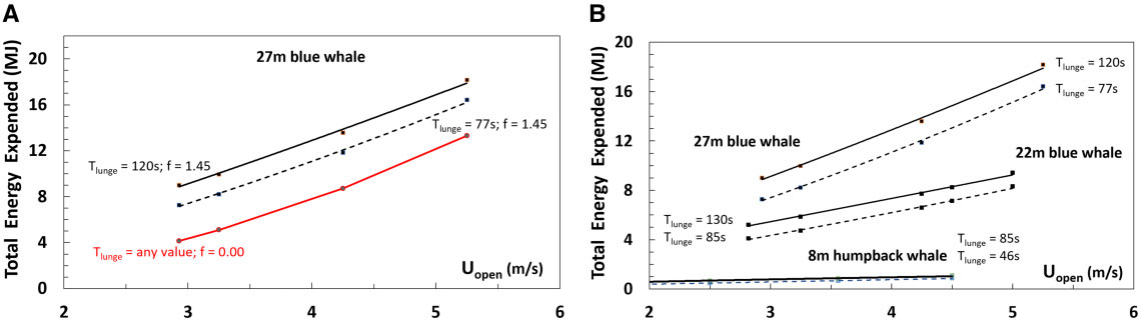
Calculated expenditures scaling versus speed and lunge duration (dots) and trend lines (continuous and dashed). (**A**) Assessment of the ceteral term in Equation (9) for the 27 m blue whale, with *f *=* *0 versus *f = *1.45. (**B**) Body size assessment with *f* =1.45 for the three showcased in the Tables, with the continuous lines corresponding to data fits: *E_1-lunge_* ∼*2.43 $U_{\text{open}}^{1.20}$* (27 m blue whale), ∼*1.75 $U_{\text{open}}^{1.03}$* (22 m blue), and ∼*0.36 $U_{\text{open}}^{0.70}$* (8 m humpback); and the dashed lines to: *E_1-lunge_* ∼*1.59 $U_{\text{open}}^{1.40}$* (27 m blue), ∼*1.12 $U_{\text{open}}^{1,24}$* (22 m blue), and ∼*0.20 $U_{\text{open}}^{0.96}$* (8 m humpback; calculated with shape drag).

Equations (2), (6), and (9) yield total expenditures as functions of the mouth-open speed when used along with the duration scaling laws just discussed. Figure 6A and B show the results for the three whales showcased in Tables 1, save for *U_open_*, and over the speed range suggested by Fig. 2. Given the lack of scaling for lunge duration (Fig. 5), two curves were generated to show likely variation within tag-informed duration extrema.

Figure 6A shows the sensitivity of the energy’s speed-scaling trend while omitting the ceteral expenditure term (*f *=* *0), versus including it when evaluated with *f *=* *1.45 (to again ensure consistency with Equation (10) at all speeds). Leaving out the *cetER* expenditures yields the smallest expenditures and ones that are explicitly independent of *T_lunge_*. Overall, the results suggest the ceteral term adding up to 50% of the total (Table 3), thereby reducing energy sensitivity to ∼${\text{U}}_{\text{open}}^{1}$ with *f *=* *1.45, rather than ∼${\text{U}}_{\text{open}}^{2}$ when using *f *=* *0 or allowing *f* to scale like the kinetic energy as suggested by Equation (10) (Hereon the symbol “∼” corresponds to an equality up to a constant factor). Qualitatively, and with or without such a term, the energetics increases two- to three-fold over the mouth-open speed range measured by bio-logging tags (Cade et al. 2016). On the other hand, Fig. 6B shows similar effects over body sizes (with *f *=* *1.45), this time while highlighting the body size dependence introduced by the factor $\left(U_{\text{open}}{}^{2}-U_{\text{close}}{}^{2}\right)/U_{\text{open}}{}^{2}$ implicit in Equation (6).

Note that with faster speeds—which bring shorter approach and engulfment durations (Potvin et al. 2020)—come higher power outputs. In comparison to land vertebrates BMR at comparable body mass (≈ *4.1 $M_{\text{body}}^{0.75}$*), single lunge power factors of 2.98× (22 m blue whale) and 3.18× (27 m blue whale) are found at *U_open_* = 3 m/s, versus factors of 5.70× (22 m) and 6.54× (27 m) at *U_open_* = 5 m/s. Looking at a similar comparison involving the more demanding engulfment expenditure rates (Equations 6 and 7), one has factors of 11.0× (22 m) and 8.50× (27 m) at 3 m/s, versus 51.2× (22 m) and 42.2× (27 m) at 5 m/s, a nearly size-independent scaling with ∼${\text{U}}_{\text{open}}^{3}$. In land animals, maximal (aerobic) power output-to-resting levels of 20 and 40 characterize those of trained athletes and pronghorn antelopes respectively (Weibel and Hoppeler 2005).

## Discussion

### Speed and prey density dependence of the efficiency

**Fig. 7:**
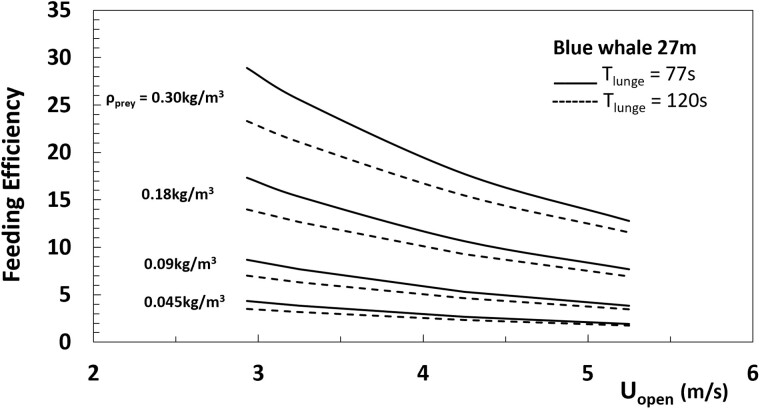
Calculated efficiency versus speed and patch prey density. Case of the 27 m blue whale listed in Table 1. Other parameters are *U_close_* from Equation (5) and *f = 1.45*.

**Fig. 8:**
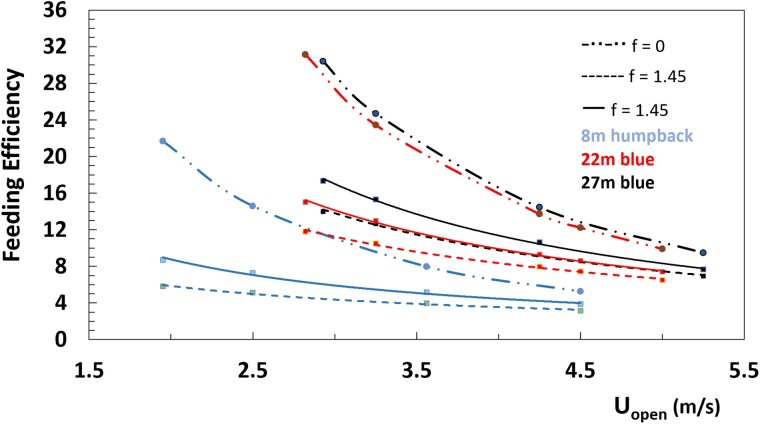
Calculated efficiency versus speed, body size and lunge duration (dots). Cases of the 27 m and 22 m blue whale and 8 m humpback whale listed in Table 1. Other parameters are *U_close_* from Equation (5), *ρ_prey_* = 0.18 kg/m^3^ and *f = 1.45*. The continuous lines correspond to data fits resulting in *FE* ∼ *79.15 $U_{\text{open}}^{-1.40}$* (27 m blue whale with *T_lunge_* = 77 s), *FE* ∼ *54.94 $U_{\text{open}}^{-1.24}$* (22 m blue; *T_lunge_* = 85 s), and *FE* ∼ *17.18 $U_{\text{open}}^{-0.97}$* (8 m humpback; *T_lunge_* = 47 s); and the dashed lines in *FE* ∼ *51.79 $U_{\text{open}}^{-1.20}$* (27 m blue; *T_lunge_* = 120 s), ∼*34.95 $U_{\text{open}}^{-1.03}$* (22 m blue; *T_lunge_* = 130 s), and ∼*9.67 $U_{\text{open}}^{-0.72}$* (8 m humpback; *T_lunge_* = 85 s). The dashed-double dot curves trace the efficiency without inclusion of the rest-of-body metabolic term (*f *=* *0), thereby scaling (exactly) as *FE ∼ M_water_/M_body_$U_{\text{open}}^{2}$*.

The expenditure scaling of Fig. 6 for the 27 m blue whale translate to the various levels of lunge efficiency *FE* shown in Fig. 7, and for prey patch densities ranging from 0.045 to 0.300 kg/m^3^. Per Equations (1) and (11), *FE* is expected to increase approximately seven-fold over the prey densities used, a result consistent with past estimates (Goldbogen et al. 2011). Rarely appreciated, however, is its sensitivity to speed for which *FE* decreases by as much as 50% over the full speed range documented by the tag data (Fig. 2). This is shown in Fig. 8 where *FE ∼$U_{\text{open}}^{-1.2}$* (27 m blue whale), *∼$U_{\text{open}}^{-1.1}$* (22 m blue) and *∼$U_{\text{open}}^{-0.80}$* (8 m humpback). Thus, and during a multi-lunge feeding dive, the lunge-to-lunge efficiency is to vary a great deal if a rorqual encounters a prey field varying widely in density, while harvesting it over a wide range of speeds (Figs. 2 and 3). As a corollary, commensurate efficiencies may be achieved whenever exploiting a low-density patch section at slow engulfment speeds versus a high density patch at high speeds.

For reasons still unknown except for the possible use of the sensory organ on the external surface of their chin (Pyenson et al. 2012), rorquals have been documented to avoid krill patches seemingly too small or tenuous (Goldbogen et al 2011; Hazen et al. 2015; Torres et al 2020), perhaps below critical density, a behavior that reflects the fact that capturing *any* number of prey items still involves engulfing the same prey-water mass at same expenditures, but at a widely varying efficiency (assuming same maximal lowering of the mandibles [∼78^°^; Fig. 1])_._ By extension, and to the extent these whales approach near-stationary krill patches at high speeds like raptors rather than at low speed like grazers, such independence on captured energy density becomes an advantage in comparison to single-prey item raptorial feeders for which prey quality is crucial and probability for success decreases with larger, more energy dense but less numerous prey (Heller and Milinski 1979; Gill and Hart 1994; McQuaid 1994; Embar et al. 2014).

### Body size dependence of the efficiency

The body size and speed variations of the efficiency at fixed prey density (ρ_prey_ = 0.18 kg/m^3^) are shown in Fig. 8, which also highlights the effects of the uncertain ceteral expenditure terms (*f *=* *0 vs. 1.45). Not surprisingly, *FE* is larger without this term, a result of the lower associated expenditures (Fig. 6A), but also scaling more steeply with speed, that is, with the inverse of the kinetic energy. Here the sensitivity to body size follows the allometric scaling of the engulfed mass ratio, that is, as *FE ∼ M_water_/M_body_$U_{\text{open}}^{2}$ ∼ $L_{\text{body}}^{0.39}$/$U_{\text{open}}^{2}$* (blue) and ∼ ${\text{L}}_{\text{body}}^{0.94}$/$U_{\text{open}}^{2}$ (humpback; Kahane-Rapport and Goldbogen 2018). On the other hand, adding the ceteral term leads to shallower scaling with respect to both speed and size. Generally, the resulting body size dependence confirms the idea of large body size conferring higher efficiencies (Goldbogen et al 2019a), that is, as long as the comparison is carried out at the same speeds (Interestingly, further analysis suggests this body-size trend to *reverse* past a broad, (non-extant) limit-size of 33–40 m).

Biologging tag data show most rorquals to lunge-feed not only near *U_open_* ≈ 3 m/s where the muscle mechanical expenditures are at their lowest and efficiency at its highest (at fixed prey density), but also—and routinely—at significantly higher speeds exceeding *U_open_* ≈ 4 m/s where the work is significantly higher and *FE* lower (Fig. 2). Thus, and at fixed patch prey density, rorquals are not only likely to experience significant reductions in efficiency, but one modulated by body size: namely, from about (*FE_1-lunge_* ≡) *FE* ≈ 16 at *U_open_* = 3 m/s to *FE* ≈ 8 at *U_open_* = 5 m/s with the blue whales, but in more modest amounts at smaller size (8 m humpback; from *FE* ≈ 8 to ≈ 5). An interesting corollary is the possibility of smaller rorquals lunge-feeding at the lower speeds and doing so at the competitive efficiencies of the larger rorquals feeding at the higher speeds, for example, with a 22 m blue whale lunging at *U_open_* ∼3 m/s versus a 27 m blue whale doing so at 4 m/s or more (Fig. 8). In the end, and again at fixed prey density, this effect is likely to result in efficiency becoming broadly peaked when only plotted versus body size, for example, Goldbogen et al. (2019a).

## Concluding remarks

Past investigations have pointed to the efficiency of lunge feeding as significantly varying over prey density (Goldbogen et al. 2011, 2012) and body size (Goldbogen et al. 2019a). Our analysis now adds prey approach and engulfment speeds as other significant factors—and ones that beg the question as to why rorquals keep feeding at faster speeds despite the loss in efficiency. Further study is needed to clarify this question, perhaps by examining other metrics of feeding success, such as oxygen use management (Hazen et al. 2015), and/or the achievement of greater net energy intake (Christiansen et al. 2013) made possible by a higher number of dives enabled by high speed lunges (whenever allowed by available stored O_2_). In such a context, lunge feeding need not be performed at the highest efficiency, but perhaps only with enough feeding bouts, and at high enough efficiency in comparison to toothed cetaceans (Goldbogen et al. 2019a).

Along with the force calculations discussed in Potvin et al. (2020), the equations presented above promise to provide useful tools for separating the biological from the physical factors affecting a whale’s lunge-feeding behavior(s) and performance. Fulfilling such a promise will depend on the collection of input parameter information that has been so far missing. One is the patch prey density (*ρ_prey_*), which, per the results shown here, could constrain lunge feeding speeds (*U_open_*) to lower values to maintain high efficiency wherever the density is low, as during feeding near the surface. More generally, correlating a whale’s trajectory with actual prey density data obtained from echo sounding (Goulet et al 2019; Cade et al. 2021) would be preferable to estimation through a geographical average (Nemoto 1983), as done here and elsewhere (Goldbogen et al. 2011; Cade et al 2016; Guilpin et al 2019, 2020). This would go a long way in not only determining the efficiency on a lunge-to-lunge basis, but also in documenting the minimal patch densities that rorquals are known to avoid (Hazen et al. 2015). Patch geometry, along with dive depth, may also turn out as essential to the understanding of the duration of the purging/filtration stage (Kahane-Rapport et al. 2020), and by extension, lunge duration in relation to other expenditure metrics and environmental factors (Goldbogen et al. 2013; Hazen et al. 2015; Lesage et al 2017).

Finally, another parameter worth further investigation is the speed at mouth closure (*U_close_*), a necessary input for the evaluation of the contribution of shape drag in smaller rorquals for which Equation (6) is no longer valid. Being proportional to a whale’s body wetted surface area, shape drag scales with size quite differently from engulfment drag which depends on the inflated buccal cavity volume (Potvin et al. 2020). By becoming more important at small body size as a classic surface-to-volume effect, shape drag adds another source of energy dissipation that is bound to lower efficiency—no doubt adding further evolutionary pressure towards larger body size (Marx et al. 2016; Goldbogen and Madsen, 2018; Goldbogen et al. 2019a; Potvin et al. 2020).

## Appendix

### Derivations

#### Closed-mouth drag work (during prey-approach)

The work done by drag during prey approach is estimated from an expression originally devised for airships (Hoerner 1965) and later applied and cetaceans (Fish 1993, 1998; Potvin et al. 2020). After insertion of a correction factor ($\tilde{F}(U)$) to account for the heaving of the tail and head during active swimming one has:

(A1) $$F_{\text{drag}}^{\text{total}}=F_{\text{drag}}^{\text{parasite}}+kM_{\mathrm{body}}a,$$

(A2) $$F_{\text{drag}}^{\text{parasite}}=\frac{1}{2}\rho_{w}S_{\text{wet}}C_{D}U^{2},$$

(A3) CD=F˜(U)[0.072Re⁡1/5]⋅[1+1.5(wmax⁡Lbody)3/2+7.0(wmax⁡Lbody)3]U2.

The equations’ input parameters are connected to the various sources of drag, namely, viscous friction, near wake pressure, turbulence, and acceleration reaction. They are as follows: seawater density and kinematic viscosity (***ρ_water_***, *ν*), instant swim speed (*U(t)*), body wetted area (*S_wet_*), drag coefficient (*C_D_*), body length and maximal diameter (*L_body_, w_max_*), Reynolds number (*UL_body_/ν*), tail heaving amplification factor ($\tilde{F}$), body acceleration (*a*), and acceleration reaction coefficient (*k; =* 0.059, 0.045, 0.036, and 0.029 for *w_max_/L_body_* = 5, 6, 7, and 8).

The work done by this drag force is estimated with a calculation assuming constant-acceleration (*a*) kinematics along a straight line; namely, as *a = (U_2_ - U_1_)/T_approach_* with the initial (*U_1_*) and final (*U_2_*) speeds, and corresponding duration *T_approach_* (as approximately suggested in Fig. 1). With the temporal velocity profile *U(t)* thus known, the travel increment *dx* used in the calculation of the work becomes *dx = U(t)dt*, a useful substitution where forces are mainly speed- and acceleration-dependent, and the speeds finite and bounded. The calculation of the work becomes an integral over time of the forces given by Equations (A1), (A2), and (A3) as follows:

(A4) $$\begin{array}{c} W_{D}=\int_{\text{initial}}^{\text{final}}\left(F_{\mathrm{drag}}{}^{\text{parasite}}+F_{\mathrm{drag}}{}^{\text{ar}}\right)dx=\int_{\text{initial}}^{\text{final}}\left(F_{\mathrm{drag}}{}^{\text{parasite}}+kM_{\mathrm{body}}\frac{dU}{dt}\right)U(t)dt\\ =\int_{\text{initial}}^{\text{final}}\left(F_{\mathrm{drag}}{}^{\text{parasite}}\right)U(t)dt+k\frac{1}{2}M_{\mathrm{body}}\left(U_{2}{}^{2}-U_{1}{}^{2}\right) \end{array}.$$

Integrating the parasite drag times *U(t)* involves the following integral:

(A5) $$\begin{array}{c} \int_{0}^{T_{\text{approach}}}\frac{U{\left(t\right)}^{2}}{U{\left(t\right)}^{0.2}}U(t)dt=\int_{0}^{T_{\text{approach}}}{\left(at+U_{1}\right)}^{2.8}dt\\ =\frac{1}{3.8a}\left(U_{2}{}^{3.8}-U_{1}{}^{3.8}\right) \end{array}.$$

The end-result is:

(A6) $$\begin{array}{c} W_{D}{}^{\text{closed}\;\text{mouth}}=\frac{1}{2}kM_{\mathrm{body}}\left(U_{2}^{2}-U_{1}^{2}\right)+\\ \tilde{F}\frac{1}{2}\rho S_{\mathrm{wet}}\frac{0.072}{{\left(R_{e{\;\text{at}\;U}_{2}}\right)}^{0.2}}\left[1+1.5{\left(\frac{w_{\max}}{L_{\mathrm{body}}}\right)}^{3/2}+7.0{\left(\frac{w_{\max}}{L_{\mathrm{body}}}\right)}^{3}\right]\frac{T_{\text{approach}}}{3.8\left(U_{2}-U_{1}\right)}U_{2}^{0.2}\left(U_{2}{}^{3.8}-U_{1}{}^{3.8}\right) \end{array}$$

#### Shape drag work during engulfment

Again assuming 1-dimensional kinematics, the work by the shape drag enabled by frictional drag, near wake drag and the acceleration reaction is obtained as follows. Writing the work as a time average yields:

(A7) $$\begin{array}{c} W_{\text{drag}}^{\text{shape}}=\int_{t=0}^{t=T_{\mathrm{engulf}}}F_{D}^{\mathrm{shape}}dx=\int_{t=0}^{t=T_{\mathrm{engulf}}}F_{D}^{\mathrm{shape}}\frac{dx}{dt}dt\\ \begin{array}{ccc} & & =\int_{t=0}^{t=T_{\mathrm{engulf}}}F_{D}^{\mathrm{shape}}U(t)dt\equiv T_{\mathrm{engulf}}\langle F_{D}^{\mathrm{shape}}U\rangle \end{array} \end{array},$$

with symbol 〈G(t)〉corresponding to the time-average of observable *G(t)*. The next step consists in writing the time average $\langle F_{D}{}^{\text{shape}}U\rangle$ into a product of time averages. The shape drag component associated with viscous friction and near-wake pressure drag will generally scale as the product of the (expanding) wetted area, times the whale’s speed squared (${\text{F}}_{D}^{\text{shape}}$∝*S_wetted_$U_{\text{whale}}^{2}$*). On the other hand and about a decelerating and inflating cavity, the additional component associated with the acceleration reaction scales according two terms: namely, a negative “decelerative” term written as the product of the deceleration times the mass of the fluid trapped in the cavity (${\text{F}}_{D}^{\text{shape}}$ ∝*a_whale_ M_water_*; with *a_whale_* < 0); and most importantly, to a second and positive “expansion” term proportional to the speed and rate of cavity expansion (∝*U_whale_ dM_water_/dt* ∼ with both factors > 0). (See the parachute examples discussed in Wolf 1974; Potvin 2008 and references therein). With the frictional drag factors *S_wetted_* increasing and *U^2^* decreasing over the same interval, and assuming both “decelerative” and “expansion” acceleration reactions partially canceling into a smaller near-constant value, it is suggested that $\langle F_{D}{}^{\mathrm{shape}}U\rangle \approx \langle F_{D}{}^{\mathrm{shape}}\rangle \langle U\rangle$, with$\langle U\rangle \approx 1/2\left(U_{\mathrm{open}}+U_{\mathrm{close}}\right)$after approximating the deceleration as constant (Fig. 1). The final step involves using the correponding time-averaged drag written as (Potvin et al. 2020):

(A8) $$\langle F_{D}{}^{\mathrm{shape}}\rangle =M_{\mathrm{body}}\frac{U_{\mathrm{open}}-U_{\mathrm{close}}}{T_{\text{engulf}}}-\langle F_{D}{}^{\mathrm{engulf}}\rangle ,$$

that is, an expression which retains implicitly contributions from frictional, near-wake and acceleration reaction drag. Per the averaged engulfment drag formula derived by Potvin et al. (2020):

(A9) $$\langle F_{D}{}^{\mathrm{engulf}}\rangle =\langle \frac{d}{dt}\left(M_{\mathrm{water}}(t)U_{\mathrm{water}}(t)\right)\rangle =\frac{\left(M_{\mathrm{water}}U_{\mathrm{whale}}\right)|{}_{@T_{\text{engulf}}}}{T_{\text{engulf}}}=\frac{M_{\mathrm{water}}U_{\mathrm{close}}}{T_{\text{engulf}}}$$

merging the above yields:

(A10) $$W_{D}^{\text{shape}}=T_{\mathrm{engulf}}\left\langle F_{D}^{\mathrm{shape}}U\right\rangle \approx T_{engulf}\langle F_{D}^{shape}\rangle \langle U\rangle =T_{\mathrm{engulf}}\left[M_{\mathrm{body}}\frac{U_{\mathrm{open}}-U_{\mathrm{close}}}{T_{\text{engulf}}}-\frac{M_{\mathrm{water}}U_{\mathrm{close}}}{T_{\text{engulf}}}\right]\cdot \frac{1}{2}\left(U_{\mathrm{open}}+U_{\mathrm{close}}\right)$$

This result is used in the work-energy theorem for this stage, namely, 12Mbody(Uopen2−Uclose2)=WDengulfment+WDshape, to result in Equation (3).
